# Size distribution of function-based human gene sets and the split–merge model

**DOI:** 10.1098/rsos.160275

**Published:** 2016-08-03

**Authors:** Wentian Li, Oscar Fontanelli, Pedro Miramontes

**Affiliations:** 1 The Robert S. Boas Center for Genomics and Human Genetics, The Feinstein Institute for Medical Research, Northwell Health, Manhasset, NY, USA; 2 Departamento de Matemáticas, Facultad de Ciencias, Universidad Nacional Autónoma de México, Circuito Exterior, Ciudad Universitaria, México 04510 DF, México; 3 Bioinformatics Group and Interdisciplinary Center for Bioinformatics, University of Leipzig, Haertelstrasse 16–18, 04107 Leipzig, Germany

**Keywords:** gene family sizes, gene set sizes, power-law, beta rank function

## Abstract

The sizes of paralogues—gene families produced by ancestral duplication—are known to follow a power-law distribution. We examine the size distribution of gene sets or gene families where genes are grouped by a similar function or share a common property. The size distribution of Human Gene Nomenclature Committee (HGNC) gene sets deviate from the power-law, and can be fitted much better by a beta rank function. We propose a simple mechanism to break a power-law size distribution by a combination of splitting and merging operations. The largest gene sets are split into two to account for the subfunctional categories, and a small proportion of other gene sets are merged into larger sets as new common themes might be realized. These operations are not uncommon for a curator of gene sets. A simulation shows that iteration of these operations changes the size distribution of Ensembl paralogues and could lead to a distribution fitted by a rank beta function. We further illustrate application of beta rank function by the example of distribution of transcription factors and drug target genes among HGNC gene families.

## 1. Introduction

A narrowly defined gene family within a genome [1,2] refers to all working genes, thus not pseudo-genes, that were produced by duplication events [3,4]. Genes in such a gene family share sequence similarity owing to their common origin, and are called paralogues [5,6]. The role of such a defined gene family in functional biology, however, is less clear as paralogues may diverge in functions [7–9]. Alternatively, a group of genes can be connected in a variety of other ways, such as similar expression patterns [10], participating in the same biology pathway, carrying out similar biological process, contributing to the same phenotype, sharing a common motif, etc. These groupings of genes can be called gene sets [11–14]. Genes in a gene set, besides being paralogues or homologues, can be based on a relationship with respect to physical proximity, chemical interaction, biological function and phenotype or medical condition [14].

To clearly distinguish gene families created by gene duplication and those by grouping using any other possible criteria, here we use the term ‘paralogues’ and ‘gene sets’ to refer to the two situations, and ‘gene family’ when such a distinction is not important. The size of paralogues in a genome can be quite uneven, with some large ones but more small ones. If *S* denotes the number of genes (size) in a paralogue, and *f*(*S*) the number of paralogues with size *S*, *f*(*S*) as a function of *S* has a power-law-like trend with negative exponents [15]. This statistical trend for paralogue sizes has been confirmed by other publications [16,17]. Similar trends have been extended to the protein universe for folds, domains, clusters, families and superfamilies [18–23].

Many theoretical and mathematical models have been proposed to explain the observed paralogue size distribution, with gene duplication [3] as the essential mechanism [24–34]. Meanwhile, biological details on paralogue evolution have been accumulated [35–44], making a realistic modelling possible. The robust and ubiquitous power-law gene and protein family size distribution is hailed as one of the laws of genome evolution [45,46].

There are three goals in this paper: (i) although the long-tailed size distribution of paralogues is well established with sufficient understanding, most analyses, with some exceptions [47], were focused on bacterial genomes. Here, we examine the human paralogues in more detail. (ii) For gene families not constructed by homology, which we call gene sets, we show that power-law rank function is not a good fitting model. A more flexible function called beta rank function can be used [48,49]. This greatly expands our toolbox in a quantitative analysis of gene families. (iii) Just as duplication operation is essential for the observed power-law distribution of paralogue sizes, we examine the effect of a combined operation of splitting and merging of gene sets. We show that these operations lead to a deviation from the power-law distribution, thus justifying the introduction of new types of fitting function such as the beta rank function.

## 2. Methods and data

### 2.1. Homology-based human gene family (paralogues) data

We use the Ensembl family (in human) which has a stable ID beginning with ENSFM [50]. The number of genes within a family (paralogue) is counted by the number of distinct Ensembl Gene IDs beginning with ENSG that share the same family ID. The data are obtained from the Ensembl Biomart (www.ensembl.org/biomart) [51] by selecting the following boxes: (i) Ensembl genes 82 in ‘choose database’; (ii) *Homo sapiens* genes (GRCh38.p3) in ‘choose dataset’; (iii) filtering ‘region (chromosome)’: 1–22,X,Y; (iv) filtering ‘gene (gene type)’: protein_coding and (v) filtering ‘gene (multi species comparisons)’: paralogous human genes.

### 2.2. Biological function-based human gene sets data

We use the Human Gene Nomenclature Committee (HGNC) gene families (www.genenames.org/cgibin/genefamilies/) [52,53]. The broad guideline for gene families (which we call gene sets here) is that they ‘signify groups of genes related by function, by sequence or by phenotype caused’ [54]. Although the hierarchical organization of gene sets is advocated [54], many high-level concepts for gene (super)families do not have an HGNC family ID. The number of genes in a gene set is counted by the number of gene names with the same gene family name or ID. Non-coding genes, RNAs, microRNAs and genes without an approved name are not counted. We examine the effect of including or excluding pseudo-genes.

### 2.3. Inverse power-law rank function and Zipf’s law

Suppose there are *n*_F_ gene families (paralogues or gene sets); these gene families are ranked from large (with many genes in a family) to small (few genes in a family), with the rank *r*=1,2,…*n*_F_. The gene family size *S*_r_ as a function of rank *r* can be called a rank-size distribution. The Zipf’s law or Pareto’s law uses an inverse power-law function: $\log(S_{r})=c-a\log(r)$ to fit the data, in log–log scale [55,56]. In the linear regression framework, the relation can be written as $\log(S_{r})∼\log(r)$ with the purpose of minimizing the error between expected (from the regression line) and observed in $\log(S_{r})$ scale. Without the log–log scale, that fitting *S*_r_=*C*/*r*^*a*^ is to minimize the error between expected and observed in the original *S*_r_ scale. The two regressions may result in different fitting parameters. It is well known that the power-law Zipf’s law *S*_r_=*c*/*r*^*a*^ corresponds to a power-law probability density function *f*(*S*)∝1/*S*^1/*a*+1^ [57], as the two are connected by a switching of *x* and *y* axes, and a derivative [58].

### 2.4. Beta rank function

The beta rank function [48,49,58–60] is the following functional form for ranked data:

2.1 $$S_{r}=C\frac{{\left(n_{F}+1-r\right)}^{b}}{r^{a}}$$

with two free parameters (*a* and *b*, where *c* is constrained by the normalization condition). In the regression framework, there are again two versions. One is a multiple linear regression in log–log scale: $\log(S_{r})=c+b\log(n_{F}+1-r)-a\log(r)$ or $\log(S_{r})∼\log(n_{F}+1-r)+\log(r)$. The second is a nonlinear regression *S*_r_∼(*n*_F_+1−*r*)^*b*^/*r*^*a*^. The multiple linear regression aims at minimizing predicted–observed error in the $\log(S_{r})$ scale, whereas the nonlinear regression minimizes that in the *S*_r_ scale. The beta rank function becomes a power-law rank function when *b*=0.

### 2.5. Measuring fitting performance of a rank function by mean-squared error and Pearson’s correlation

The fitting performance of a function/regression can be measured in different ways. We use two measures here: (i) mean-squared error (MSE):

2.2 $$\mathrm{MSE}=\frac{1}{n_{F}}\sum_{r=1}^{n_{F}}{\left(O_{r}-E_{r}\right)}^{2},$$

where ‘observed’ *O*_r_ can either be *S*_r_ or $\log(S_{r})$ and ‘expected’ *E*_r_ can either be regression-predicted *S*_r_ or $\log(S_{r})$. The quantity without the normalization factor (1/*n*_F_) is called sum of squared errors (SSE), or residual sum of squares (RSS), or sum of squared residuals (SSR). (ii) Pearson’s correlation coefficient between *O*_r_ and *E*_r_,

2.3 $$r_{\mathrm{OE}}=\frac{\sum_{r=1}^{n_{F}}(E_{r}-{\bar{E}}_{r})(O_{r}-{\bar{O}}_{r})}{\sqrt{\sum_{r=1}^{n_{F}}{\left(E_{r}-{\bar{E}}_{r}\right)}^{2}\sum_{r=1}^{n_{F}}{\left(O_{r}-{\bar{O}}_{r}\right)}^{2}}}$$

again in either the *S*_r_ scale or $\log(S_{r})$ scale. The coefficient of determination *R*^2^, used in, e.g. Martínez-Mekler *et al.* [49], is the square of *r*_*OE*_ [61], p. 29:

2.4 $$R^{2}=r_{\mathrm{OE}}^{2}=\frac{{\left(\sum_{r=1}^{n_{F}}(E_{r}-{\bar{E}}_{r})(O_{r}-{\bar{O}}_{r})\right)}^{2}}{\sum_{r=1}^{n_{F}}{\left(E_{r}-{\bar{E}}_{r}\right)}^{2}\sum_{r=1}^{n_{F}}{\left(O_{r}-{\bar{O}}_{r}\right)}^{2}}.$$

(iii) Kolmogorov–Smirnov (KS) distance between the cumulative distribution function (cdf) from observed data and cdf from the fitted values. KS distance in cdf is defined as the maximum distance between the two cdfs in the cumulative probability axis. That axis is equivalent to the rank axis in a rank–frequency plot, normalized by the maximum rank.

### 2.6. Comparing the fitting performance of two functions with different number of fitting parameters by Akaike information criterion and Bayesian information criterion

This part is essential identical to page 185 of Venables & Ripley [62] and the appendix of Li & Miramontes [59]. If we assume the difference between observed and fitted values follow a normal distribution at all ranks, the likelihood of the fitting function is

2.5 $$L=\prod_{i=1}^{n_{F}}\frac{e^{-{\left(O_{i}-E_{i}\right)}^{2}/2\sigma^{2}}}{\sqrt{2\pi \sigma^{2}}}=\frac{e^{-\sum_{i}^{n_{F}}{\left(O_{i}-E_{i}\right)}^{2})/2\sigma^{2}}}{{\left(2\pi \sigma^{2}\right)}^{n_{F}/2}},$$

where the variance of the noise can be estimated from the data: ${\hat{\sigma }}^{2}=\mathrm{MSE}$.

The Akaike information criterion (AIC) [63] is defined as: $\mathrm{AIC}=-2\log\hat{L}+2p$, where $\hat{L}$ is maximized likelihood, *p* is the number of parameters in the statistic model and Bayesian information criterion (BIC) [64] defined as $\mathrm{BIC}=-2\log\hat{L}+p\log n_{F}$. Replacing *σ* by the estimated $\hat{\sigma }$, we obtained the maximized likelihood, which after log is

2.6 $$\log(\hat{L})=C-\frac{n_{F}}{2}\log({\hat{\sigma }}^{2})=C-\frac{n_{F}}{2}\log(\mathrm{MSE})$$

then

2.7 $$\mathrm{AIC}=n_{F}\log(\mathrm{MSE})+2\cdot p+\mathrm{const}.$$

and

2.8 $$\mathrm{BIC}=n_{F}\log(\mathrm{MSE})+\log(n_{F})\cdot p+\mathrm{const}.$$

The fitting function with the lowest AIC/BIC is a better function [65].

### 2.7. Measuring fitting performance of a rank function by empirical *p*-value through simulation

Following Clauset *et al*. [57], we compare the distance between the observed data and fitted function with the distances generated from re-samplings (or bootstraps [66]). The re-sampling uses the true function to generate data, and the produced distances are supposed to be noise. When the observed distance tends to be larger than those from the re-sampling, we may question the validity of the fitted function. The proportion of re-sampling distances larger than the observed one is called empirical *p*-value. Then, a smaller empirical *p*-value can be used to reject the fitted function being a good function for this data [57] (see also, e.g. the appendix of Moreno-Sánchez *et al*. [67]).

We carry out these steps: (i) fitting a ranked data by a ranking function (e.g. Zipf’s function or beta rank function equation (2.1)); (ii) use the fitted rank function to construct an empirical cdf, noting that rank plot can be directly converted to an empirical cumulative plot [58]. We may write *y*=*cdf*(*x*) with *y*∈(0,1); (iii) randomly sampling *y*s from the uniform distribution in (0, 1), and through the empirical cdf, obtain a simulated dataset which is generated by the fitted rank function (*x*=*cdf*^−1^(*y*)). The *x*s are rounded off to integers, and integer *x*=1 is discarded as we require gene families to have at least one member; (iv) the simulated values *x*s are ranked and fitted by a fitted function (same functional form as the one used in the observed data, e.g. Zipf’s law or beta rank function). We use the KS distances as the distance measure between the data and the fitted function. KS distance is the maximum difference between the observed and fitted points in the *y*-direction. The KS distances between the simulated set and the corresponding fitted function are calculated; (v) repeat steps ii–iv. The proportion of KS distances from re-sampling replicates larger than the KS distance from the original observed data is the empirical *p*-value.

### 2.8. Computer program used

We use the R statistical package (www.r-project.org), where the function *lm* is used for linear regression, *cor.test* for correlation coefficient, *ks.test* for KS distance, *approxfun* for empirical cdf, etc.

### 2.9. List of transcription factor genes

A list of 1987 transcription factor (TF) genes is obtained from the supplementary table 2 of Vaquerizas *et al*. [68], with the URL: http://www.nature.com/nrg/journal/v10/n4/extref/nrg2538-s3.txt.

### 2.10. List of drug target genes

A list of 2829 drug target genes is obtained from the IUPHAR/BPS (International Union of Basic and Clinical Pharmacology/ British Pharmacological Society) Guide to PHARMACOLOGY site: http://www.guidetopharmacology.org/DATA/targets_and_families.csv [69].

## 3. Results

### 3.1. Human paralogue sizes are characterized by the power-law distribution reasonably well

**Figure 1.**
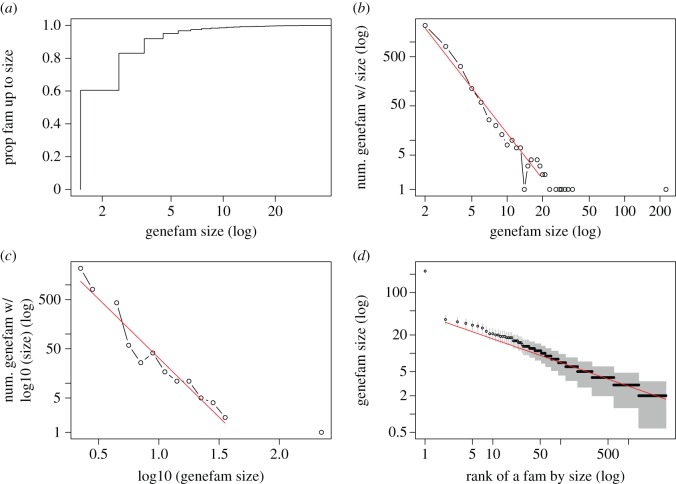
Distributions of human paralogue sizes from Ensembl. (*a*) Cumulative distribution. (*b*) Histogram (in log–log scale). (*c*) Histogram of log-transformed gene family sizes (*y*-axis in log scale). (*d*) Rank–frequency distribution (ranked individual paralogue sizes). A possible error bar is drawn in grey colour.

By the procedure described in the Method section, 3586 gene families (paralogues) are produced from Ensembl. By definition, a gene family should contain at least two genes. There are 2168 (60.5%) two-gene families, 810 (22.6%) three-gene families and 318 (8.9%) four-gene families. These ‘nuclear families’ already make up more than 90% of all families, as shown in the cumulative distribution (figure 1*a*). The fall off of the number of size-*S* gene families as a function of *S*, when shown in double logarithmic scales in figure 1*b*, is roughly a straight line, indicating that the histogram is a power-law function. Similarly, the histogram can be obtained over the log(*S*), as in figure 1*c*, with *S* in log scale, which is again a straight line.

The distribution of human paralogues can be viewed in the rank–frequency plot (figure 1*d* in log–log scale). Besides the largest gene family, ENSFM00250000000002, a zinc finger family with 225 genes, other points in figure 1*d* can be reasonably approximated by a power-law function (i.e. Zipf’s law). Note that the second, third and fourth largest gene families, ENSFM00730001521153 (36 genes), ENSFM00730001521252 (33 genes) and ENSFM00760001714593 (31 genes), are also zinc fingers.

While figure 1 shows qualitatively that the paralogues follow a power-law-like distribution, we would like to examine whether it is good quantitatively. The MSE of the three fitting lines in figure 1*b*–*d* is 0.107, 0.255, 0.010; and the *R*^2^ and 95% CIs are 0.973 (0.922–0.991), 0.943 (0.804–0.984), 0.936 (0.931–0.940). All these measures are calculated in the scale in where the regression was carried out (i.e. log–log scale in figure 1*b*,*d*, linear–log in figure 1*c*). If the largest paralogue ENSFM00250000000002 is included in a fitting, then all fitting performances go down: MSEs are 1.08, 0.79, 0.011, *R*^2^s and 95% confidence intervals are 0.767 (0.552–0.888), 0.843 (0.992–0.952), 0.933 (0.929–0.937).

The fitting of points with non-zero values in figure 1*b* leads to an estimation of the exponent *α* in log⁡(p)∼−αlog⁡(S) to be 3.1. The estimation of *α* from figure 1*d* leads to 1/0.389+1≈3.6. The difference between the two estimations is perhaps due to the slightly different scalings at the two ends: in figure 1*b*, smaller paralogues contribute more to the fitting, whereas in figure 1*d*, individual large paralogues contribute more to the parameter fitting.

We use the re-sampling approach described in the Method section to assess the fitting of Zipf’s law. Of the 10 000 replicates, 9692 have larger KS distances with their own fitted function than the KS distance from the observed data, and 46 have equal KS distances. This leads to an empirical *p*-value of 0.97, indicating that Zipf’s law is a very good fitting function of the observed data. Another indirect confirmation is by drawing an error bar ($\pm \sqrt{y}$) [70] for the data point in figure 1*d*. The shaded area enveloped by error bars maintains the straight line trend.

### 3.2. Curated human gene family sizes are better characterized by the beta rank function

We compile a list of 749 gene families from HGNC, with 717 contain at least two genes. ‘Not approved’ genes and RNAs (including microRNA, tRNA, piRNA, etc.) are excluded. A gene might be present in multiple families, either because the gene can have multiple functions or a gene can be viewed from different perspectives in the construction of the gene families. Including the size-1 families, there are 19 461 entries in the gene-genefamily file, with 15 181 genes appearing once, and 1931 genes appearing in multiple families. The largest five families are C2H2-type zinc fingers (ZNF; 714 without counting pseudo-genes and 720 if pseudo-genes are included), Solute carriers (SLC) (390/396), cluster of differentiation molecules (CD) (389/394), ring finger proteins (RNF) (263/275) and WD (or WD40 for 40 tryptophan–aspartic amino acids)-repeat-domain containing (WDR) (261/262).

**Figure 2.**
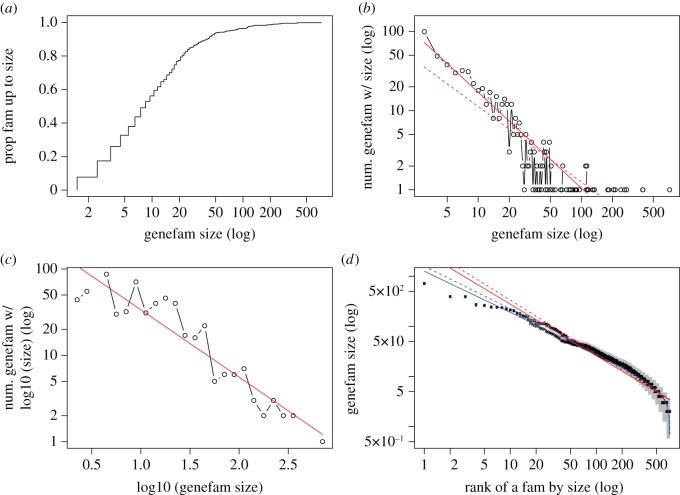
Distributions of gene set sizes from the HGNC. (*a*) Cumulative distribution. (*b*) Histogram (solid line is a linear regression fitting for points *y*>1, and dashed line is that for all points). (*c*) Histogram of log-transformed gene family sizes. (*d*) Rank–frequency distribution (crosses and solid lines: excluding pseudo-genes; circles and dashed lines: including pseudo-genes). Red fitting lines are Zipf’s laws, blue fitting lines are beta rank functions. A possible error bar is drawn in grey colour.

Various distributions for the HGNC gene families are shown in figure 2. The empirical cdf of gene family sizes in figure 2*a* shows that unlike the Ensembl paralogues, most HGNC families are not ‘nuclear families’. It is reasonable, because curating a gene family with only two or three genes is less useful for practical purposes. The histogram of gene family sizes in figure 2*b* may seem to point to a power-law distribution. However, fitting all points in figure 2*b* with *y*>0 (dashed line, slope=−1.02) and fitting only points with *y*≥2 (solid line, slope=−1.23) are not consistent with each other. On the other hand, a coarse-graining of the large families by using the log size before fitting with power-law function seems to work better (figure 2*c*, slope=−1.79). The discrepancy with the Zipf’s power-law becomes more pronounced if we plot the ranked individual gene family sizes (figure 2*d*). Including pseudo-genes (circles) versus excluding pseudo-genes (crosses) exhibits some difference in the middle rank ranges.

The power-law or Zipf’s law’s fitting performances of figure 2*b*–*d* are as follows (for figure 2*b*, points with *y*≥2 are used): MSE is 0.158, 0.217, 0.079; *R*^2^ and 95% CI is: 0.856 (0.752–0.919), 0.876 (0.728–0.946), 0.933 (0.925–0.944); KS distance is: 0.156, 0.130, 0.173. These fitting performances are worse than those in figure 1*b*–*d*. In figure 2*b*, if points with *y*=1 are also used, MSE=0.378, *R*^2^=0.765 (0.667–0.838), then the fitting is much worse. In figure 2*d*, if pseudo-genes are considered, MSE=0.070, *R*^2^=0.935 (0.925–0.944), then fitting is very similar to the fitting performance when pseudo-genes are excluded.

We use the same re-sampling procedure to test the performance of Zipf’s law for the whole range (for gene set sizes excluding pseudo-genes). In one round of re-sampling with 10 000 replicates, two of the KS distances between the randomly generated data and their fitting function are larger than the observed one. This 0.0002 empirical *p*-value indicates that Zipf’s law is a poor fitting function. The shaded area in figure 2*d* enveloped by error bars also shows that straight lines do not fit the data well.

It is a common practice to dismiss the tail that does not conform to a power-law as finite-size effects, based on the assumption that we do not have enough samples to observe the true value [71]. Although we may gradually improve the fitting performance of Zipf’s law by removing the smaller families (empirical *p*-values are 0.01, 0.16, 0.20, 0.48 if we only fit family sizes larger than or equal to 8, 9, 10, 11), we actually remove common events by doing so, not rare events as implied in the idea of finite-size effects.

Not only could the finite-size-effect argument not justify smaller gene sets being rare events, we also have a general belief that all points in the rank–frequency plot such as figure 2*d* should be fitted, and not to use that argument of finite-size effects arbitrarily ([72]). Previous studies illustrate great potential of the beta rank function in fitting observational data [48,49]. The fitted parameter values in equation (2.1) are *a*=0.85 and *b*=0.34 by linear regression of log size over log-rank (*a*=0.82 and *b*=0.32 with pseudo-genes). Because *a*>*b*, we may consider our fitting function a modification of the Zipf’s law.

Figure 2*d* shows a much better fitting of the data by the beta function (blue) than by the power-law function (red). The improvement of the fitting performance can be further quantified by MSE (0.0179 without pseudo-genes, 0.0155 with pseudo-genes) and *R*^2^ (0.985 without pseudo-genes, 0.986 with pseudo-genes). Because it is not fair to compare the fitting performance between two functions of different numbers of fitting parameters, we require that the increase of likelihood (or decrease of the fitting error) is more than compensated by the cost of using one more parameter

3.1 $$\Delta \mathrm{AIC}={\mathrm{AIC}}_{\mathrm{beta}}-{\mathrm{AIC}}_{\mathrm{zifp}}=n_{F}\log\frac{{\mathrm{MSE}}_{\mathrm{beta}}}{{\mathrm{MSE}}_{\mathrm{zipf}}}+2<0$$

or

3.2 $$\Delta \mathrm{BIC}={\mathrm{BIC}}_{\mathrm{beta}}-{\mathrm{BIC}}_{\mathrm{zifp}}=n_{F}\log\frac{{\mathrm{MSE}}_{\mathrm{beta}}}{{\mathrm{MSE}}_{\mathrm{zipf}}}+n_{F}<0$$

The first term in equation (3.1) and equation (3.2) is −1065.9 (without pseudo-genes) and −1086.4 (with pseudo-genes), proving that the rank beta function is better than the power-law function.

### 3.3. Simulating a beta rank function by a split–merge model

We propose a simple model to explain rank functional form such as equation (2.1) which describes the sizes of curated gene sets. We first assume gene set sizes to initially follow a power-law distribution. A curator of the gene sets may fine-tune the collection by (i) splitting the larger (and the second largest) gene sets into two smaller ones and (ii) merging 6% of randomly chosen sets into larger ones by a two-to-one operation. The reason for not choosing the smaller gene sets, but any gene sets, is that once we chose a threshold gene set size to decide which one is small, a discontinuity is introduced to the distribution.

The motivation for the split operation is that curators of gene family databases may find subtle functional difference between genes in the largest families. The motivation for merging gene families could be due to a realization of a common function between two families. These operations are distinctly different from duplication which is an intrinsic mechanism to grow gene family sizes. Our two operations can be considered to be extrinsic ones imposed by curators.

**Figure 3.**
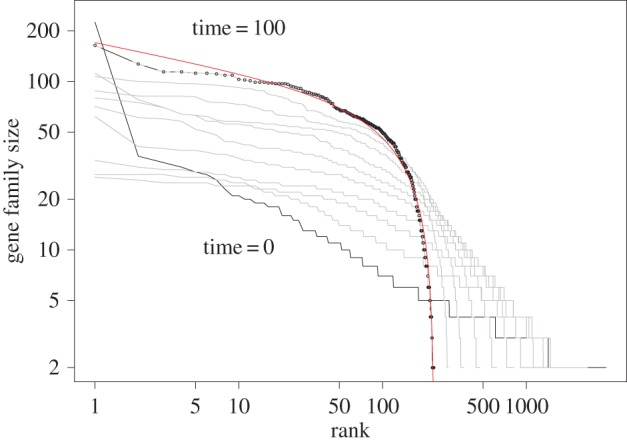
Dynamics of gene set sizes. The initial gene set sizes are taken from the Ensembl paralogues. At each round, the largest gene set is split randomly into two sets, and 6% of all gene sets are merged by a two-to-one operation. The ranked gene set sizes are plotted after every 10 rounds, until reaching the 100th round. The fitting of the 100th round gene set sizes by the rank beta function leads to *a*=0.16 and *b*=0.84.

We use the paralogues from Ensembl as the starting gene sets. The ranked gene set sizes after every 10 rounds of operation according to our dynamic model are plotted in figure 3. At the 100th round, the ranked gene set sizes are fitted by a beta rank function (solid line in figure 3). Visual impression indicates that beta rank function fits well the data points at the 100th step. Simulation with other initial distributions has also been carried out, such as uniform distribution, absolute normal distribution, lognormal distribution, chi-square distribution, etc. The resulting distributions after 100 or so iterations are all similar, although there are subtle differences. We also observe that the *b*>*a* in equation (2.1) holds true in our simulation of this particular model.

Because the initial gene set sizes, which are the sizes of paralogues, should be a consequence of duplication and deletion [15] or duplication and mutation [24], the sizes of gene sets produced by our dynamic model is a consequence of all these operations: duplication, deletion, mutation, splitting and merging, though their relative contribution to the final distribution is unclear. Besides modelling of gene family sizes, there has been theoretical modelling of power-law distribution on various other entities in genomes [73–85]. Although these works do not model gene family sizes directly, the connection between a mechanism (duplication) and a feature of the distribution (power-law) is unmistakable. We expect the extra mechanisms discussed here could be a cause of deviating the power-law distribution in these entities also.

## 4. Discussion and conclusions

### 4.1. Gene family size information is important for enrichment tests

Gene set is an indispensable part of a microarray expression analysis [13,86–89]. A typical question is whether the overlapping genes between a gene set with a specific function and a list of differentially expressed genes (between two phenotypes) from microarray data are more than expected by chance. If such an overlap is unlikely by chance, measured by the *p*-value from a statistical test on enrichment, one may infer the relevance of that gene set to the phenotype under study. Because the test result depends on the size of the gene set, enrichment results of two different gene sets may not be comparable. In Subramanian *et al.* [13], gene sets with size *S* smaller than 15 are not considered, and enrichment score is normalized by a permutation-obtained mean value which is gene-set-size-dependent. Thus, it is important to characterize the distribution of all gene sets and one should be aware of the gene set size before reaching conclusion on the relevance of a gene set.

### 4.2. Distribution of transcription factor genes among gene families

The beta rank function equation (2.1) used in figure 2*d* expands our ability to fit gene set size data. Here, we show two more examples to illustrate its flexibility. We examine how human TF genes are clustered in different HGNC gene sets. Towards this, we use the 1987 TFs listed in Vaquerizas *et al.* [68], and search them in the HGNC gene sets. Some HGNC gene sets are clearly exclusively TFs. For example, in the C2H2 zinc finger family (ZNF), 633 out of 720 genes are TFs; in the basic helix–loop–helix family (BHLH), 96 out of 110 are TFs; all 52 genes in HOXL subclass homeoboxes and 47 out of 49 nuclear hormone receptors (NR) are TFs, etc.

**Figure 4.**
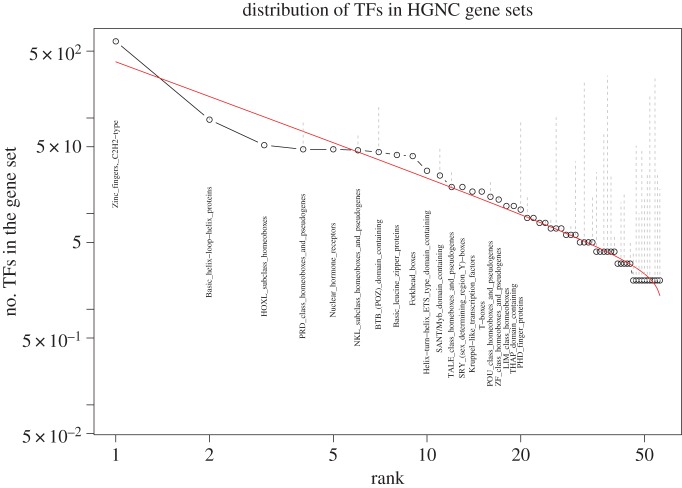
Ranked number of transcription factors (TFs) in individual HGNC gene families (in log–log scale). Gene family name is marked for the top 20 families. The gene family sizes (including both TFs and other non-TF genes) are represented by dots and are linked with the number of TFs by dash lines.

Figure 4 shows the ranked distribution of TFs in HGNC gene sets (families) when there is at least two TFs in a set. There are 1389 TFs satisfying the condition (in 56 families). Some gene sets may only contain a few TF genes, with the majority being non-TFs (the original gene set sizes are linked to the number of TFs in that gene set in dashed lines in figure 4). A beta rank function is used to fit the data. Re-sampling with 10 000 replicates, 1618 with KS distance larger than observed, and 2180 replicates with the same KS distance as observed, leads to an empirical *p*-value of 0.38. As a comparison, an empirical *p*-value using the power-law rank function is 0.12.

### 4.3. Distribution of drug target genes among gene families

A similar analysis is carried out for the pharmacological or drug target genes [69]. We use the target list obtained from the The International Union of Basic and Clinical Pharmacology/British Pharmacological Society (IUPHAR/BPS) Guide to PHARMACOLOGY site, which only includes those with a UniProtKB/Swiss-Prot ID, thus also having an HGNC name. The first groups of targets annotated in detail include G protein-coupled receptors, ion channels and nuclear hormone receptors [90]. Many proteins labelled as receptor, channel, transporter, kinase, etc. are drug targets or target candidates.

**Figure 5.**
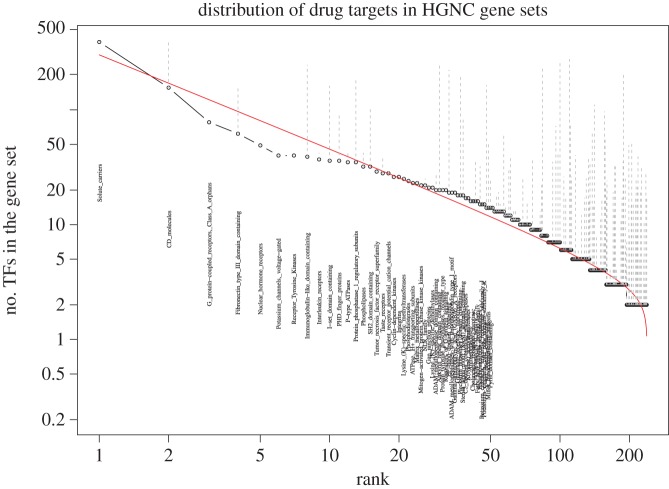
Ranked number of drug target (DT) genes in individual HGNC gene families (in log–log scale). Gene family name is marked for the top 50 families. The gene family sizes (including both DTs and other non-DT genes) are represented by dots and are linked with the number of DTs by dash lines.

Figure 5 shows the distribution of these target genes in the HGNC gene families. The majority of genes in the solute carrier gene family (390/396) are IUPHAR/BPS targets. Solute carriers are transporters that transport a large variety of smaller molecules such as ions, amino acids, neurotransmitters, sugars, etc, and are known to be good candidates for drug targets [91]. G protein-coupled receptors (GPCRs) reside on the cell membrane (pass through it seven times), sense molecular signals from outside the cell and initiate signal transduction inside the cell. As a result, GPCRs are also candidates for drug targets [92]. Although CD molecules or cell surface molecules provide the second most numerous targets, 60% of CD proteins are still not listed as targets. As seen in figure 5, the beta rank function provides a qualitative trend of the distribution of drug target genes in the HGNC gene families. The systematic deviation from the fitting curve in figure 5 is very similar to that for TFs (figure 4), even though the gene families involved in the two examples are completely different.

### 4.4. The effect of including pseudo-genes on gene family sizes

**Figure 6.**
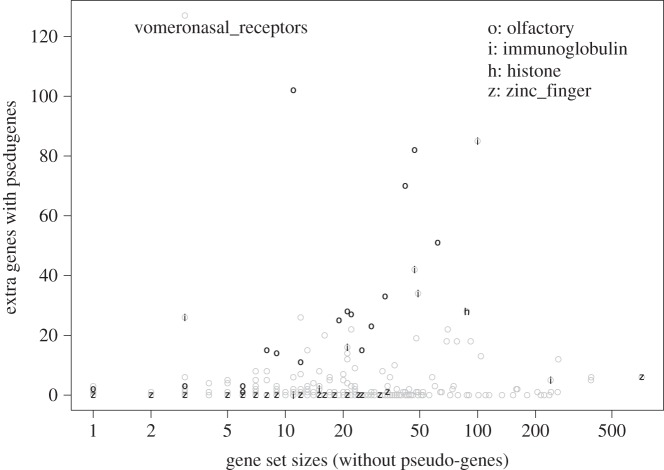
Increase of gene set size with the inclusion of pseudo-genes (plotted against the gene set size excluding pseudo-genes, in log scale). Olfactory (o), immunoglobulin (i), histone (h) and zinc finger (z) gene families are marked. The inclusion of pseudo-genes has great impact on olfactory and histone gene families, whereas minimum impact on zinc finger gene families.

There are more than 10 000 pseudo-genes in the human genome [93]. As most pseudo-genes do not have any known function, only a small proportion of them show up in the HGNC gene families/sets. Excluding pseudo-genes reduces the total number of entries in the gene sets by 1396. In figure 6, we plot the increase of gene set size due to the inclusion of pseudo-genes, as a function of the gene set size. Pseudo-genes have the most dramatic impact on olfactory gene sets [94,95], as expected because the reduced importance of sense of smell to human survival leads to a larger amount of disabling mutations in these genes. The gene set vomeronasal receptors, which is related to the olfactory sense, is labelled individually: it contains three genes without pseudo-genes, whereas 130 genes with pseudo-genes are included. Pseudo-genes also cause large increase of size for immunoglobulin gene sets. On the other hand, pseudo-genes have either no or very little impact on zinc finger gene sets.

In conclusion, we aim at bridging two aspects of biological fields, evolutionary biology and functional biology (as well as human biomedical research), by comparing two types of gene families: paralogues and gene sets. While the size distribution of paralogues is well studied and its understanding is through the dynamics of duplication and deletion/mutation, the size distribution of gene sets has rarely been studied and its functional form was unknown. We observed that the size distribution of functional gene sets does not need to follow a power-law distribution, and its deviation from the power-law can be fitted by beta rank function. We propose a mechanism to drive the size distribution away from a power-law function, by splitting the largest gene sets and by randomly merging a small proportion of other gene sets. These results are potentially useful in a gene enrichment analysis as gene set size affects a gene enrichment test result.

## Data accessibility

The data files used in figures 1, 2, 4 and 5 are available at http://dx.doi.org/10.5061/dryad.rc1kv.
